# Successful treatment of recurrent epididymo-orchitis: Laparoscopic excision of the prostatic utricle

**DOI:** 10.4103/0971-9261.54813

**Published:** 2009

**Authors:** Ashish Jiwane, S. V. S. Soundappan, John Pitkin, Daniel T. Cass

**Affiliations:** Department of Academic Surgery, The Children's Hospital at Westmead, The University of Sydney, Australia

**Keywords:** Epididymo-orchitis, hypospadias, laparoscopy, prostatic utricle

## Abstract

Prostatic utricle presenting with recurrent epididymo-orchitis is not uncommon. Excision of prostatic utricle is the treatment of choice. The various techniques described in literature suffer from the disadvantages of incomplete excision due to poor view. We report the successful laparoscopic excision of prostatic utricle in childhood.

## INTRODUCTION

Prostatic utricle is present in 14% of cases with proximal hypospadias. While majority are asymptomatic, excision is performed when they are large and or symptomatic. We describe our experience with laparoscopic excision of the utricle.

## CASE REPORT

**Figure 1:**
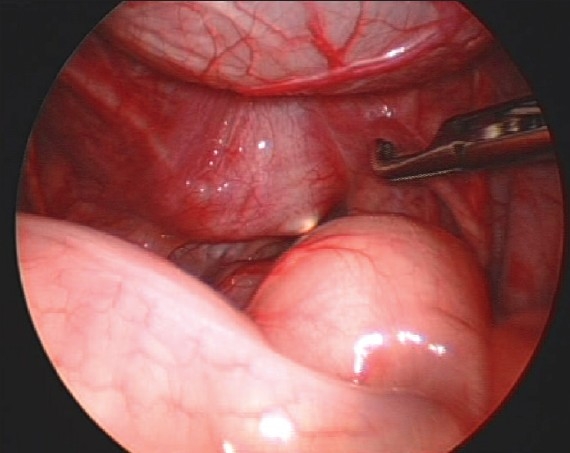
The laparoscopic view of the prostatic utricle with light of the cystoscope

A 2-year-old boy who previously treated for hypospadias was evaluated for recurrent left epididymo-orchitis. He had a diverticulum of the neourethra, seen as a large prostatic utricle on ultrasound and micturating cystourethrogram [Figure 1]. The urethral diverticulum was excised, and the large prostatic utricle was managed conservatively. He continued to have recurrent epididymo-orchitis; hence, he underwent a laparoscopic excision of the utricle and has since been asymptomatic for last 8 months.

### Technique

**Figure 2:**
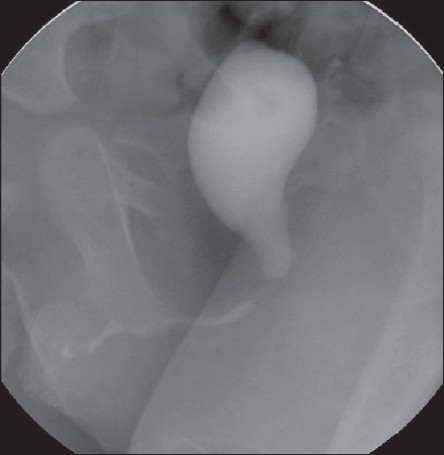
MCU showing the huge prostatic utricle, the catheter has entered into the utricle. Bladder is not seen

Urethroscopy was done and the scope was left in the utricle. Laparoscopy was performed using 3 ports, 5mm umbilical port (for camera) was placed by open Hassan technique and two lateral 5-mm operating ports. The cystoscope was used as a guide for dissection [Figure 2]. Complete excision was achieved using diathermy and sharp dissection. A 2/0 PDS endoloop was used to ligate the neck of the utricle. The cyst was removed via the lateral port.

## DISCUSSION

The prostatic utricle is an enlarged diverticulum in the posterior urethra of males. It was first described by Englisch in 1874.[1] It is an embryological remnant resulting from a transient decline in the fetal testicular function during the critical period of urethral formation in the 9^th^-10^th^ week of fetal life. In males, the mullerian structures regress in response to the mullerian inhibiting substance (MIS). The cyst arises from the mullerian duct and/or decreased androgenic stimulation of the urogenital sinus.[2,3]

The true incidence of prostatic utricle is not known; it reportedly is present in 14% of patients with proximal hypospadias and in 57% of patients with perineal hypospadias.[2]

Ikoma *et al*. proposed a classification of the prostatic utricle depending on the urethrographic configuration into four types from grade 0 to grade III.[4]

Most prostatic utricles are asymptomatic. Symptoms are related to the size and can present with infection, stones, recurrent epididymitis, incontinence, infertility and neoplastic degeneration.[1,2,4–6] They can present at any age, with more than a third presenting in children.[7]

Neoplastic degeneration has been reported in 3% of the prostatic utricles with a peak incidence in the 4^th^ decade of life.[8] Early excision is, hence, recommended in symptomatic patients.

Several surgical approaches have been described for the excision, including the abdominal extravesical, transvesical transtrigonal, suprapubic, perineal, transrectal anterior or posterior sagittal approaches and endoscopic fulguration.[2,5,6,9–14] These procedures can be difficult and involve complications. The suprapubic, retrovesical and transvesical approaches are met with incomplete excision in 58% of the cases. The perineal route was reported to be successful in only 43%. Transperineal or transrectal cyst aspiration and endoscopic manipulation resulted in a 35% recurrence rate.[7] There appears to be no satisfactory method for the successful and complete excision of the prostatic utricle. Ligation of vas has been reported to relieve the patient of epididymo-orchitis, but in our view, it does not address other complications and risk of neoplastic degeneration of enlarged utricle.[15]

Laparoscopic technique for the excision of prostatic utricle in children was first reported by Yeung *et al.* in 2001.[1] They reported successful excision of prostatic utricle in four boys. A median follow up of 11 months did not show any UTI's in their patients. Another successful laparoscopic excision of symptomatic prostatic utricle was published by Willets in 2003.[7] The advantages of the laparoscopic technique are reported as 1) clear view of the deep pelvic structures 2) good cosmesis 3) enabling examination of the rest of the abdomen and urogenital system 4) complete excision.[1,7] Laparoscopic excision under cystoscopic guidance is an effective technique offering good view and easy dissection. The use of cystoscopic light source aids in identification of the utricle and dissection. The scope can be used as a retractor to help in laparoscopic dissection.

Our case reaffirms the advantages of laparoscopic surgery in these deep pelvic anomalies. We recommend that laparoscopic excision with cystoscopic guidance should be used as the mode of treatment for troublesome, symptomatic prostatic utricles.

## References

[1] Yeung CK, Sihoe JD, Tam YH, Lee KH (2001). Laparocopic excision of prostatic utricles in children. BJU Int.

[2] Devine CJ, Gonzalez Serva L, Stecher JF, Devine PC, Horton CE (1980). Utricular configuration in hypospadias and intersex. J Urol.

[3] Husmann DA, Allen TD (1997). Endoscopic management of infected enlarged prostatic utricle and remnants of rectourethral fistula tracts of high imperforate anus. J Urol.

[4] Ikoma F, Shima H, Yabumoto H (1985). Classification of enlarged prostatic utricle in patients with hypospadias. Br J Urol.

[5] Lima M, Morabito A, Libri M, Bertozzi M, Dómini M, Lauro V (2000). Laparoscopic removal of a persistent mullerian duct in a male: Case report. Eur J Pediatr Surg.

[6] Monfort G, Guys JM (1981). Transvesical approach to surgery on the prostatic utricle. Chir Pediatr.

[7] Willets IE, Roberts JP, McKinnon AE (2003). Laparoscopic excision of prostatic utricle in child. Pediatr Surg Int.

[8] Schuhrke TD, Kaplan GW (1978). Prostatic utricle cysts (Mullerian duct cysts). J Urol.

[9] Meisheri IV, Motiwale SS, Sawant VV (2000). Surgical management of enlarged prostatic utricle. Pediatr Surg Int.

[10] Ritchey ML, Benson RC, Kramer SA, Kelalis PP (1988). Management of mullerian duct remnants in the male patient. J Urol.

[11] Keramidas DC, Kapouleas GP, Papandreou E (1995). The posterior sagittal approach for the excision of a prostatic utricle cyst. Br J Urol.

[12] Siegel JF, Brock WA, Pena A (1995). Transrectal posterior sagittal approach to prostatic utricle. J Urol.

[13] Kuhn EJ, Skoog SJ, Nicley ER (1994). The posterior sagittal pararectal approach to posterior urethral anomalies. J Urol.

[14] Rossi F, De Castro R, Ceccarelli PL, Dòmini R (1998). Anterior Sagittal transanorectal approach to the posterior urethra in pediatric age group. J Urol.

[15] Ynai T, Okazaki T, Yamataka A, Urao M, Kobayashi H, Kato Y (2005). Cysts of the ejaculatory system: A report of two cases. Pediatr Surg Int.

